# Solitary Confinement: Surprising Post‐Copulatory Behaviour of an Australian Species of Wishbone Spider (Mygalomorphae: Anamidae: *Aname*)

**DOI:** 10.1002/ece3.73606

**Published:** 2026-05-03

**Authors:** Andrea Piccinini, Jeremy D. Wilson, Mark S. Harvey, Michael G. Rix, Kimberley S. N. Wong, Leigh W. Simmons

**Affiliations:** ^1^ Centre for Evolutionary Biology, School of Biological Sciences The University of Western Australia Crawley Western Australia Australia; ^2^ Collections and Research Western Australian Museum Perth Western Australia Australia; ^3^ Biodiversity and Geosciences Program Queensland Museum Collections and Research Centre Brisbane Queensland Australia; ^4^ Centre for Biosecurity and One Health Murdoch University Murdoch Western Australia Australia

**Keywords:** Araneae, copulation, mate guarding, mating systems, sexual selection

## Abstract

Among invertebrates, spiders are regarded as a model group for sexual selection studies. However, our understanding of their mating behaviour is highly biased towards species in the infraorder Araneomorphae. Knowledge of the mating systems of mygalomorph spiders (infraorder Mygalomorphae) remains rudimentary at best. Here, we report on the mating behaviour of a recently described Australian wishbone spider (Anamidae: *Aname inexpecta*) from south‐western Western Australia. In doing so, we present the first detailed observations of mating behaviour of a member of the family Anamidae and describe a remarkable, novel male sexual behaviour for mygalomorph spiders (‘burrow plugging’). This behaviour involves the male collecting soil with his chelicerae and placing it over the entrance to the female's burrow, sealing the entrance before actively guarding the plugged retreat. We provide some of the first insights into post‐copulatory mate guarding in burrowing mygalomorph spiders, contributing to our understanding of mating systems in a group where such studies are rare.

## Graphical abstract

Araneomorph spiders are regarded as a model group for sexual selection studies, while the mating system of mygalomorph spiders remains largely unknown. We here report on the mating behaviour of an Australian wishbone spider species, involving a novel post‐copulatory behaviour for mygalomorph spiders (‘burrow plugging’). This observation also provides some of the first insights into post‐copulation mate guarding in a group where such behavioural studies are rare.

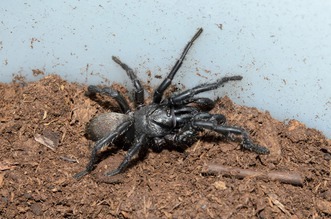

## 1 Introduction

Spiders are well known for their many adaptations arising from sexual selection, including extreme sexual dimorphism, nuptial feeding, sexual cannibalism and exaggerated prosomal modifications (e.g., Eberhard 2004; Huber 2005; Kuntner and Coddington 2020; Kralj‐Fišer et al. 2025). Hence, spiders are regarded as model systems for studies of ecology and evolution (Eberhard 2004; Huber 2005; Herberstein 2011).

Our understanding of sexual selection in spiders is highly biased towards species in the infraorder Araneomorphae, while courtship and mating behaviours in mygalomorph spiders (infraorder Mygalomorphae) and segmented spiders (suborder Mesothelae) are currently poorly understood (Ferretti et al. 2013). This lack of knowledge has resulted in the assumption that mygalomorphs have very simple sexual behaviours compared to araneomorphs (e.g., Gerhardt 1929; Platnick 1971). However, several recent studies have challenged this paradigm by revealing complex behaviours in mygalomorph spiders, documenting premating courtship behaviours which may involve tactile‐chemical communication and vibratory or seismic signals (e.g., Ferretti et al. 2013; Ghirotto and Guadanucci 2020; Nicoletta et al. 2022; Frank et al. 2023; Rendall et al. 2026).

Seismic signals are likely the main communication channel in burrowing mygalomorph species, and they involve many actions carried out by males once they reach the retreat of the female or the silk lines associated with it: body vibrations, palpal drumming and leg tapping are the most common actions among a diverse suite of behaviours (Pérez‐Miles and Perafán 2017). Adult males of many species also bear modified structures on leg I or II, called claspers or spurs. Although assumed to clasp or brace the female during copulation, the precise mechanics of these structures probably vary widely across the infraorder, and are generally unknown (Ferretti et al. 2013; Frank et al. 2023). However, their male‐specific expression and divergence across species suggest that sexual selection may be responsible for their evolution (Coyle 1985; Jackson and Pollard 1990; Ferretti et al. 2013).

Sexual selection in spiders can act at several stages during the reproductive process, from male sperm induction to post‐copulation mate choice (Schneider and Andrade 2011). One of the most investigated post‐copulatory mechanisms of sexual selection in araneomorph spiders is female mate choice (Huber 2005). Post‐copulatory sexual selection has been found to act via the internal morphology of the female reproductive tract, a mechanism known as cryptic female choice (Thornhill 1983; Eberhard 1996; Eberhard and Cordero 1995). Female genitalia bear paired sperm‐storage organs called spermathecae, where sperm is stored after males insert their emboli in an alternating fashion during copulation (Pérez‐Miles and Perafán 2017). There is evidence that, in polyandrous females, post‐copulatory processes can control the transfer and utilisation of sperm and influence which individual male, or males, achieve paternity of the offspring (Burger et al. 2003; Eberhard 2004). In this context, the first or last male to mate often sires the majority of offspring (e.g., Schneider and Andrade 2011; Matzke et al. 2022), although this is not universally the case (Tuni et al. 2020).

*Aname* L. Koch, 1873 is a burrowing mygalomorph spider genus endemic to Australia, currently containing 140 described species (World Spider Catalog 2026). Ongoing research estimates that the total number of *Aname* species is likely to exceed 300, possibly making it the most diverse mygalomorph spider genus in the world (Wilson, Harvey, et al. 2025). These spiders, commonly called Australian wishbone spiders, have colonised a wide variety of habitats throughout the Australian continent, including heathlands, arid and semi‐arid woodlands, deserts and transitional forests (Harvey et al. 2018; Rix et al. 2021). The males of this genus bear a spur with a terminal megaspine on the tibia of leg I that is assumed to be used for holding and positioning the female while mating (Harvey et al. 2018). However, very little is known about the natural history of *Aname*, and no information on sexual behaviour is currently available for this or any other genus belonging to the Australian endemic family Anamidae Simon, 1889. Even within the clade Nemesioidina, which includes Anamidae, our understanding of mating behaviour rests on a few key laboratory studies, on the species *Acanthogonatus centralis* Goloboff, 1995 and *Xenonemesia platensis* Goloboff, 1989 (two South American Pycnothelidae), and *Diplura macrura* L. Koch, 1841 (a Brazilian Dipluridae) (Ferretti et al. 2011, 2012; Ghirotto and Guadanucci 2020; Opatova et al. 2020; Montes de Oca et al. 2022).

In this note, we report on the mating and post‐copulatory behaviour of *Aname inexpecta* Piccinini et al. 2025, a recently described wishbone spider species that builds open burrows in open eucalypt forests in the Northern Jarrah Forest subregion of south‐western Western Australia (Piccinini et al. 2025). This work is the first to present detailed observations on the mating behaviour of an anamid spider and provides some of the first insights into post‐copulatory mate guarding in burrowing mygalomorph spiders.

## 2 Materials and Methods

The *Aname inexpecta* individuals used in this study were collected by burrow excavation in March 2025 from their natural Jarrah [ *Eucalyptus marginata* Sm]/Marri [ *Corymbia calophylla* (R.Br.) K.D.Hill & L.A.S.Johnson] and Wandoo [ *Eucalyptus wandoo* Blakely] eucalypt forest habitats at two separate sites north‐east of Bannister, Western Australia (WA): one male and two females were obtained from the same population (POP1) at 32°31′S, 116°29′ E, while a third female was obtained from a second population (POP2) at 32°30′S, 116°29′ E, ~2.1 km from POP1. The geographic coordinates are rounded to the nearest minute to minimise the risk of poaching or live trading (Marshall et al. 2022; Lassaline et al. 2023). The four specimens were kept in captivity under natural lighting and temperature conditions at The University of Western Australia (UWA), Perth, WA. The females were kept individually in deep buckets (30 cm length × 30 cm width × 25 cm height) filled with peat moss substrate, allowing the spiders to burrow in semi‐natural conditions; the male was kept in a shallower container (35 cm length × 15 cm width × 12 cm height), due to the reduced burrowing capacity of adult males. The females were also offered crickets [ *Teleogryllus oceanicus* (Le Guillou, 1841)] as prey every week. Once all mating experiments were finished, all individuals were euthanised for taxonomic purposes (Piccinini et al. 2025) and accessioned into the arachnological collection of the Western Australian Museum (WAM), Perth, where a unique WAM registration number was assigned to each specimen: WAM T169962 (♂), WAM T169963 (♀), WAM T169964 (♀), WAM T169965 (♀).

Mating observations were carried out in a Controlled Temperature Room (CTR) with a set temperature of 22°C and a 12 h light/dark cycle. Due to the nocturnal activity of the spiders, all the CTR artificial lights were turned off, apart from a small red lamp placed above the female burrow.

**FIGURE 1:**
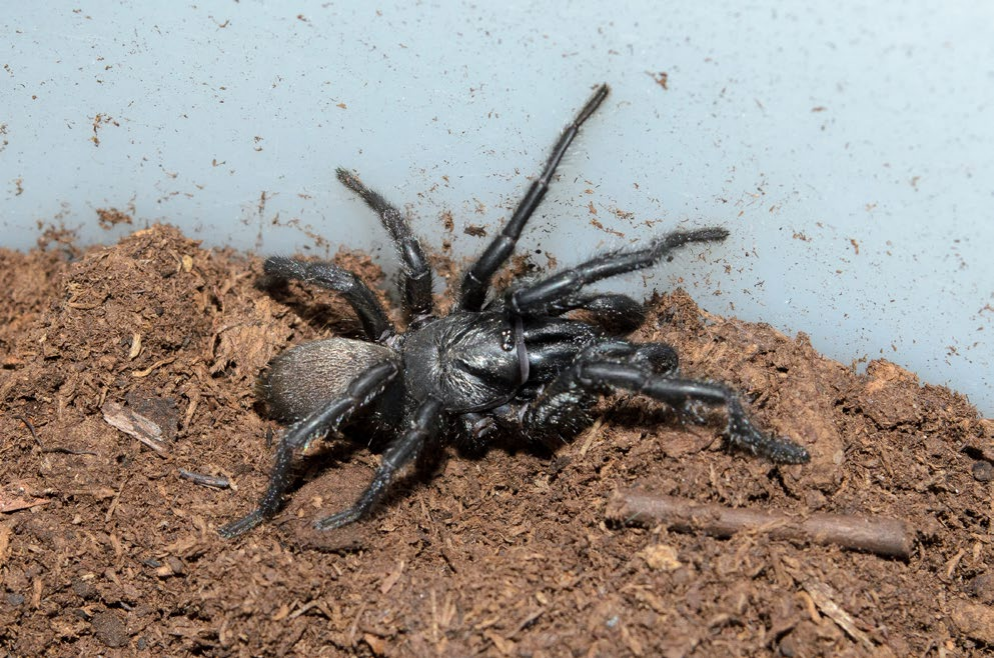
Post‐copulatory mate guarding in *Aname inexpecta*: Male positioned directly above the entrance of the female burrow, previously plugged with substrate (‘burrow plugging’), 17 h 54 m after the end of the mating encounter. Image by A. Piccinini.

**FIGURE 2:**
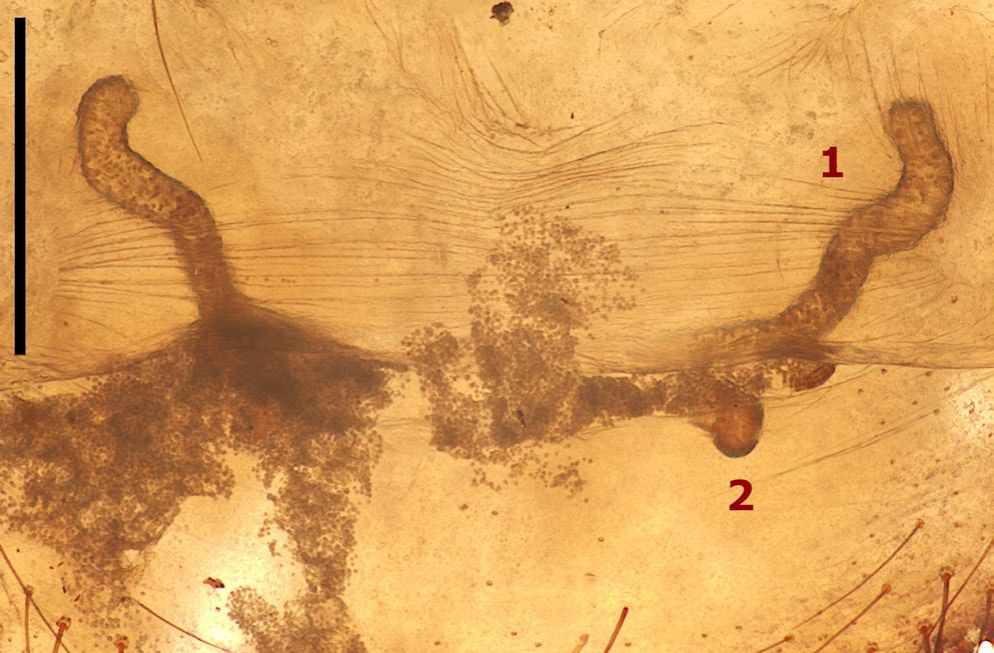
Internal genitalia of the mated female of *Aname inexpecta* (WAM T169963). (1) Lateral vesicle of left spermatheca. (2) Medial vesicle of left spermatheca. Sperm granules deposited by the male can be seen coming out of the lateral vesicles. Scale bar = 0.5 mm.

At the start of each observation, the male was placed into the enclosure of a female, away from her burrow, and the subsequent behaviour of the pair was recorded. Encounters were filmed and photographed using a Nikon 18–55 mm lens mounted on a Nikon D5100 APSC reflex camera, either handheld or placed on a tripod. When possible, the observed behaviours were described according to the terminology of Ferretti et al. (2013). The clips were visualised, edited and trimmed in Adobe Premiere Pro 2025 version 25.2. Figure 1 was taken with the same camera and lens setup used for the videos, and Figure 2 was created using GIMP version 2.10.38 (https://www.gimp.org/).

## 3 Results

### 3.1 Courtship and Mating Behavioural Sequence

A total of seven mating trials were staged; however, only one resulted in a successful copulation. In the remaining trials, the spiders were either inactive or the female proved unreceptive despite the male's advances. Most behaviours here described can be seen in Video S1, and the frequency and duration of the observed behaviours are presented in Table 1.

**TABLE 1:** Data on number (*n*), frequency (bouts per second, *b/s* ± standard deviation, SD) and duration in seconds (*s* ± SD), for sexual behaviours observed and measured in *Aname inexpecta*. Note that number, frequency and duration of the male spasmodic beats refer to the movements of only one leg, due to the absence of the male right leg II.

Behavioural unit	Number (*n*)	Frequency (*b/s*)	Duration (*s*)
Palpal boxing ♂	13	8.17 ± 1.36	5.03 ± 1.64
Leg II spasmodic beats ♂^a^	7	5.13 ± 0.53	4.40 ± 1.01
Pre‐copulatory body jerks ♀	86	1.18 ± 0.87	0.85 ± 0.47
Mating ♀ ♂	1		1080.37
Lunging ♂	1		0.62
Post‐copulatory body jerks ♀	14	2.38 ± 0.63	0.42 ± 0.14
Leg tapping/palpal drumming ♂	6	9.30 ± 2.14	0.97 ± 0.19
Burrow plugging ♂	6		11.46 ± 4.20

^a^ Number, frequency and duration refer to the movements of only one leg.

The successful mating encounter started with the placement of the male in the female enclosure, away from her retreat. The male remained motionless for around 13 min, until the female came to the top of her burrow and performed semi‐circular movements around the entrance, presumably spinning silk. This behaviour most likely initiated courtship: in fact, the male started moving and entered the burrow of the female, head‐first, around 35 s later. The first stages of the courtship behaviour took place inside the burrow, and so the exact behaviours for this portion of courtship remain unknown.

After approximately 75 s, the male and female emerged from the burrow, in close interaction. At this stage, the pair were facing each other, with the male outside the burrow entrance and the female half inside, with her first two pairs of legs protruding outside. The male proceeded to clasp the female's mouthparts with both first legs; however, due to poor lighting conditions, it was not possible to observe the specific point of contact between the male's tibia I spur and megaspine and the female. This precopulatory clasping behaviour did not involve raising the female's body.

Upon clasping the female, the male performed intermittent palpal boxing coincident with gentle, fast spasmodic beating movements of the second pair of legs, while clinging on the ground with the fourth legs. This last behaviour had the effect of partially pulling the female out of the burrow. Palpal boxing involved very fast and alternated movements of the male pedipalps, reaching towards the sternum of the female. The spasmodic leg beats consisted of gentle and rapid elevations and extensions of the male's left leg II, which beat and scraped the female's right leg II. Palpal boxing was performed 13 times, and the spasmodic beats were performed seven times, always simultaneously with palpal boxing, with the boxing lasting longer than the leg movements. The female performed intermittent body jerks 86 times; these behavioural bouts involved high amplitude twitching of her legs, either with left and right legs at the same time or one side after the other (see Table 1). Simultaneously, she appeared to hold her position inside the burrow, partially counteracting the male pulling behaviour. The whole courtship phase lasted around 6 min and 30 s (393 s).

The copulation phase also took place at the edge of the burrow entrance, still with the female almost completely inside the burrow. The male continued using the spur and megaspine on his modified tibia I to clasp the female mouthparts, lifting her into an oblique position. The male then lowered his body to ground level, positioning his anterior body into the entrance of the burrow, towards the female's abdomen, and inserted his emboli into the spermathecae while using his left leg II to hug the female. Hugging behaviour consisted of enveloping the female prosoma with his leg, thus holding her still during embolic insertion. The pattern of insertion of the emboli could not be observed or recorded, as the female kept her abdomen inside the burrow for the entire duration of the copulation. The whole mating phase lasted for approximately 18 min (1080s) and ended with the pair suddenly separating. The male appeared to commence the separation, using his legs III and IV to retract from the female and subsequently performing a very fast (0.6 s) forward movement, almost resembling an attempt to bite the cephalothorax of the female. We refer to this supposedly aggressive behaviour as ‘lunging’ (see Video S1). At this stage, the male was still using his first pair of legs to clasp the female. The female became very active after the male's lunging behaviour, and performed high‐amplitude body jerks 13 times. The two individuals completely detached after approximately 31 s, during another sudden lunging movement by the male.

### 3.2 Post‐Copulatory Burrow Plugging

Shortly after mating ended, the male once again approached the female burrow, performing gentle seismic signals including leg tapping and palpal drumming at the entrance of the burrow. The female did not respond to these signals.

Subsequently, the male used his chelicerae to pick up soil and place it over the entrance to the burrow, thus confining the female inside (Video S1). We refer to this behaviour as ‘burrow plugging’. The male repeated the behaviour six different times, and once finished, the entrance of the female retreat was completely covered with substrate and invisible from outside. During the plugging period, the female was continuously moving inside the burrow; although it was not possible to detect or record the precise nature of these movements, as they were performed underground, the soil covering was seen to move up and down. Immediately after plugging the burrow, the male patrolled the surroundings, returning to rest above the burrow every time, and performed light tapping behaviours with his pedipalps and legs I. The post‐copulatory phase lasted around 6 min and 40 s (404 s).

Finally, the male settled directly above the plugged burrow and remained motionless. The individuals were left together overnight, and the male was found still guarding the female, sitting on top of her burrow, 17 h 54 m after the end of the mating encounter (Figure 1). Both individuals were then removed from the enclosure and euthanised for taxonomic purposes (Piccinini et al. 2025). Additionally, the genital plate of the mated (WAM T169963) female was dissected; the internal genitalia were characterised by sperm granules coming out of the lateral spermathecal vesicles (Figure 2).

## 4 Discussion

This study reveals the reproductive behaviour of *A. inexpecta* during both the pre‐copulatory and the post‐copulatory phases. The pre‐copulatory and peri‐copulatory behavioural repertoire of this species appears to be broadly similar to that described for other mygalomorph spiders (e.g., Ferretti et al. 2012, 2013; Frank et al. 2023). However, we report a remarkable post‐copulatory behaviour, never observed in mygalomorph spiders before, in which the male seals the mated female into her burrow and guards her, most likely against rival males.

Post‐copulatory burrow plugging behaviour is an indication of the potential for polyandry in *A. inexpecta*, as mate guarding is a widespread post‐copulatory strategy in spiders (Herberstein et al. 2005; Schneider and Andrade 2011) and other animals in response to sperm competition (Simmons 2001). Multiple paternity in mygalomorph spiders has been recently reported for the first time in the North American halonoproctid *Bothriocyrtum californicum* O. Pickard‐Cambridge, 1874 (Ramirez et al. 2024). This belongs to the clade Domiothelina, which is phylogenetically distant from the nemesioid family Anamidae (Opatova et al. 2020). This suggests that polyandry could be widespread across the Mygalomorphae tree of life, implying a potentially overlooked role of sexual selection in the evolution of post‐copulatory behaviours of these spiders.

Mate guarding is predicted to be particularly common in species with haplogyne female genital morphology, like mygalomorph and haplogyne araneomorph spiders (Schneider and Andrade 2011). Indeed, all haplogyne females possess shared ducts for insemination (afferent) and fertilisation (efferent), and many store sperm in a single receptaculum within each spermatheca, thus providing the opportunity for males to use their emboli to remove sperm from previous males (Peretti and Eberhard 2010). However, it should be noted that like many haplogyne spiders, species of *Aname*, including *A. inexpecta*, possess both a lateral and a medial vesicle on each spermatheca (Figure 2) (e.g., Harvey et al. 2018; Wilson, Harvey, et al. 2025; Wilson, Rix, et al. 2025; Piccinini et al. 2025), although the role of this additional genital complexity is currently unknown. Mate guarding is currently regarded as an uncommon behaviour in Mygalomorphae (Ferretti et al. 2013), with only a single reported case for *Mecicobothrium thorelli* Holmberg, 1882, a South American species of Mecicobothriidae. After mating, some males of *M. thorelli* remained in the sheet‐webs of the females for 30 min, vigorously attacking the next courting males in a ritualised manner (Costa and Pérez‐Miles 1998). Our results reveal that *A. inexpecta* exhibits a novel form of post‐copulatory mate guarding and, although no intrasexual interactions have yet been observed, we suggest that this species also likely exhibits male–male aggressive behaviour. A previous report of aggressive intrasexual interactions in mygalomorphs comes from *Porrhothele antipodiana* (Walckenaer, 1837), which males show a violent behaviour defined as grappling, often ending with one of the contenders killing and eating the other (Jackson and Pollard 1990).

Post‐copulatory mate guarding has been well‐studied in web‐building araneomorph spiders, some of which display extreme behaviours such as emasculation and genital plugging (e.g., Calbacho‐Rosa et al. 2010, 2026; Uhl et al. 2010; Kralj‐Fišer et al. 2011; Kuntner et al. 2015). However, prior to our observation, no examples of female social confinement achieved through burrow plugging were known for any mygalomorph spider species. The first pieces of evidence of post‐reproductive burrow sealing in Araneae come from *Allocosa alticeps* (Mello‐Leitão, 1944) and *Allocosa brasiliensis* (Petrunkevitch, 1910), two South American Lycosidae. These species exhibit a reversal in courtship roles, with copulation happening inside the male burrow; after mating, males donate their burrow to females, cooperating with them in sealing its entrance (Aisenberg et al. 2007; Aisenberg and Costa 2008). A similar confinement behaviour can also be found in hornbills (family Bucerotidae): in these hollow‐nesting birds, the female closes the entrance of the nest cavity with the help of the male, prior to entering and laying her eggs inside (Kozlowski et al. 2015). During this period, the female and hatchlings rely completely on the male for food provisioning (Kemp 1995). In our study, movement of the soil covering the burrow entrance indicated that the female was actively engaged while the male plugged the burrow from the outside. This suggests that, as in *Allocosa* and in hornbills, the female may be cooperating with the male to seal the entrance. However, what we observed in *A. inexpecta* could equally generate sexual conflict, if female interests are better served through multiple mating (Arnqvist and Rowe 2005).

Overall, our observations suggest that the genus *Aname* exhibits a complex behavioural repertoire, especially with regard to post‐copulatory behaviour. Specifically, the male burrow plugging in *A. inexpecta* is the first observation of confinement behaviour in Mygalomorphae. Further studies will be required to determine whether this is a cooperative behaviour or rather an indication of sexual conflict, potentially involving female polyandry and male–male competition.

## Author Contributions

**Andrea Piccinini:** conceptualization (lead), data curation (lead), funding acquisition (equal), methodology (lead), project administration (lead), writing – original draft (lead), writing – review and editing (lead). **Jeremy D. Wilson:** funding acquisition (equal), supervision (equal), writing – review and editing (equal). **Mark S. Harvey:** funding acquisition (equal), supervision (equal), writing – review and editing (equal). **Michael G. Rix:** funding acquisition (equal), supervision (equal), writing – review and editing (equal). **Kimberley S. N. Wong:** methodology (equal), writing – review and editing (equal). **Leigh W. Simmons:** conceptualization (equal), funding acquisition (equal), supervision (lead), writing – review and editing (lead).

## Funding

This work was supported by the Australian Biological Resources Study, 4‐H3KOGBR, Society of Australian Systematic Biologists, Biologic Environmental Survey, Holsworth Wildlife Research Endowment, University of Western Australia and Alcoa Foundation.

## Conflicts of Interest

The authors declare no conflicts of interest.

## Supporting information


**Video S1:** A compilation of videos of the key sexual behaviours presented by male and female of *Aname inexpecta* during our laboratory observation.

## Data Availability

The authors have nothing to report.
