# Supplementary figures and images for: Solitary Confinement: Surprising Post‐Copulatory Behaviour of an Australian Species of Wishbone Spider (Mygalomorphae: Anamidae: Aname)
Source: Ecol Evol. 2026 May 3;16(5):e73606. doi: 10.1002/ece3.73606 (PMC13135893; doi:10.1002/ece3.73606)

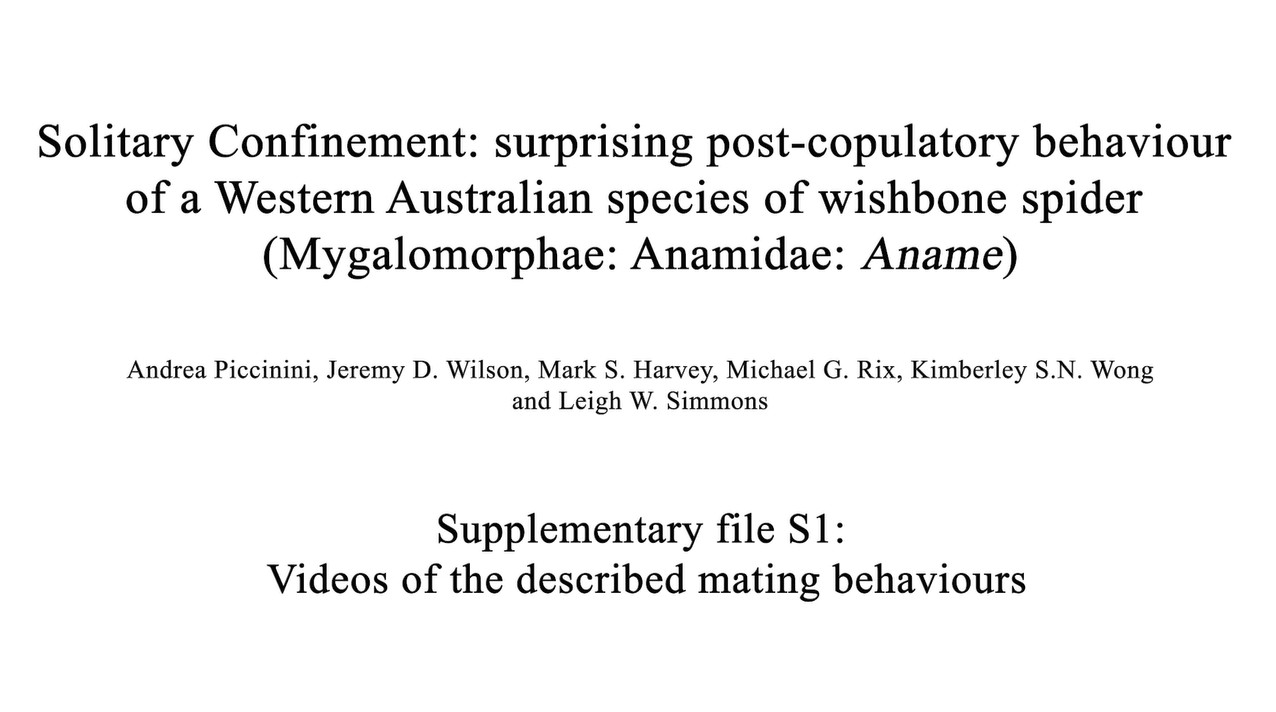

Supplement: Supplementary file 1 — Video S1: A compilation of videos of the key sexual behaviours presented by male and female of Aname inexpecta during our laboratory observation. [file ECE3-16-e73606-s001.zip › ece373606-sup-0001-Supinfo1@20260414 - Piccinini etal - 2026 - Video Placeholder Image.jpg]
